# An Observational Study on Early Empiric versus Culture-Directed Antifungal Therapy in Critically Ill with Intra-Abdominal Sepsis

**DOI:** 10.1155/2014/479413

**Published:** 2014-05-15

**Authors:** Winnie Lee, Yixin Liew, Maciej Piotr Chlebicki, Sharon Ong, Pang Lee, Andrea Kwa

**Affiliations:** ^1^Department of Pharmacy, Singapore General Hospital, Outram Road, Singapore 169608; ^2^Department of Infectious Diseases, Singapore General Hospital, Outram Road, Singapore 169608; ^3^Department of Anaesthesiology, Singapore General Hospital, Outram Road, Singapore 169608

## Abstract

*Objective.* To compare early empiric antifungal treatment with culture-directed treatment in critically ill patients with intra-abdominal sepsis. *Methods.* A prospective observational cohort study was conducted between August 2010 and July 2011, on SICU patients admitted after surgery for gastrointestinal perforation, bowel obstruction or ischemia, malignancy and anastomotic leakages. Patients who received antifungal treatment within two days of sepsis onset were compared to patients who received culture-directed antifungal treatment in terms of mortality rate and clinical improvement. Patients' demographics, comorbidities, severity-of-illness scores, and laboratory results were systematically collected and analysed. *Results.* Thirty-three patients received early empiric and 19 received culture-directed therapy. Of these, 30 from the early empiric group and 18 from culture-directed group were evaluable and analysed. Both groups had similar baseline characteristics and illness severity. Patients on empiric antifungal use had significantly lower 30-day mortality (*P* = 0.03) as well as shorter median time to clinical improvement (*P* = 0.025). Early empiric antifungal therapy was independently associated with survival beyond 30 days (OR 0.131, 95% CI: 0.018 to 0.966; *P* = 0.046). *Conclusion.* Early empiric antifungal therapy in surgical patients with intra-abdominal sepsis was associated with reduced mortality and warrants further evaluation in randomised controlled trials.

## 1. Introduction 

The incidence of nosocomial invasive mycoses has increased markedly over recent years [1], particularly in the surgical intensive care unit (SICU) where they have been associated with significant mortality and morbidity [2–7]. The predominant fungal pathogen implicated in these nosocomial mycoses is* Candida*, which was also ranked as the fourth most common cause of nosocomial bloodstream infections in United States [2, 4, 7, 8] with rates ranging from 2.1 to 20 incidences of candidemia per 1000 intensive care unit admissions [1, 6, 7, 9]. Critically ill ICU patients with invasive candidiasis (IC) have been associated with poor clinical outcomes, with crude and attributable mortality in the ranges of 20–70% and 10–40%, respectively [1, 5–7].

Definitive diagnosis of IC relies on conventional culture technique, given its nonspecific clinical signs and symptoms. Unfortunately, culture-based diagnoses are hampered by the low yield of* Candida* species in blood cultures and a slow turnaround time of 2-3 days [10, 11], leading to delayed initiation of effective antifungal therapy. As poor outcomes had been associated with delayed initiation of antifungal therapy for invasive candidiasis [12, 13], alternative early antifungal intervention strategies, that is,* prophylactic*,* preemptive*, and* empiric *therapy, for high-risk patients have been explored, in place of culture-directed antifungal therapy [2, 4, 11, 14, 15].

Antifungal prophylaxis with fluconazole had been assessed in several trials [16–18], involving a diverse spectrum of ICU and critically ill surgical patients and meta-analyses of these studies, which seemed to suggest a reduction in the incidence of invasive candidiasis but not mortality [19, 20]. The lack of a clear benefit with antifungal prophylaxis may be due to vastly different ICU patient groups with varying degrees of risk for IC and death; that is, patients with underlying hematological malignancies are at much higher risk for IC and death than patients with pneumonia. Presently, the widespread adoption of antifungal* prophylaxis* is not recommended by the latest guidelines from the Infectious Disease of America (IDSA) in ICU patients as the cost-effectiveness and potential ecologic effect of such an approach have not been fully elucidated [21].

An alternative strategy would be* preemptive* therapy where treatment is started in response to increased* Candida *riskscores, for example,* Candida *colonization index (CCI), or levels of* Candida* biomarkers such as (1-3)-*β*-D-glucan [11, 21, 22]. Combining clinical risk factors with the use of the CCI in the preemptive approach has shown negative predictive values from 95 to 99%. However, the positive predictive value is much lower, ranging from 10% to 60%.* Candida* biomarkers display higher positive predictive values; however, they lack sensitivity and are thus not able to identify all cases of invasive candidiasis [23]. Furthermore, these tests detecting the presence of fungal elements in patients' bodily fluids are often very costly, thus increasing costs to patients and/or health-care funding agencies. Hence, the logistical and financial implications of this approach may limit its utility in resource-poor healthcare facilities.

Early empiric therapy may be the most practical strategy for centers with limited resources. With this approach, there is a need to clearly identify which patient groups will benefit the most from early antifungal treatment. This is because the indiscriminate initiation of empiric antifungal agents may render them ineffective over time with the progression of fungal resistance. Patients who receive antifungal agents are also at risk of developing adverse effects due to these drugs. As such, the benefit of starting antifungal treatment must always outweigh patients' risk for developing drug-related serious adverse effects.

Known risk factors for IC include the use of broad-spectrum antibacterial agents, use of central venous catheters, receipt of parenteral nutrition, receipt of renal replacement therapy by patients in ICUs, neutropenia, use of implantable prosthetic devices, and receipt of immunosuppressive agents or gastrointestinal surgery [21]. Patients who have recently undergone single or repeated major gastrointestinal operations are at especially high risk for IC as they possess most of the risk factors listed above. Therefore, the benefit of starting early empiric antifungal treatment in these patients will probably outweigh any potential risks of experiencing drug-related adverse effects.

In our hospital, there was no consensus among the ICU and infectious diseases physicians on which was the best strategy to adopt. This led to varied practices on the treatment of fungal infections in the ICU, with some physicians subscribing to early empiric approach while others using the culture-directed approach. The preemptive strategy was not employed due to limited funds and manpower constraints.

A prospective observational cohort study was thus performed on our patients at high risk for intra-abdominal sepsis and IC, to evaluate if the early empiric use of antifungal treatment led to better survival than culture-directed treatment. Secondary objectives were to determine if early empiric antifungal therapy could shorten length of SICU and hospital stay, as well as time to clinical improvement.

This study was approved by the SingHealth Centralized Institutional Review Board (CIRB number: 2009/698/D) and a waiver of informed consent was obtained.

## 2. Study Objectives

The objectives of the study were as follows:

1. to determine if the use of early empiric antifungal treatment was associated with lower mortality when compared to culture-directed treatment;
2. to evaluate if early empiric antifungal treatment was associated with shorter ICU stay, shorter time to clinical improvement, and length of hospitalization for survivors.

## 3. Methods and Materials

### 3.1. Study Design and Patient Population

This was a single-centre, prospective observational cohort study conducted at a tertiary hospital between July 2011 and January 2012. All patients admitted to the SICU after surgery within the study period were identified from the electronic hospital records and screened for study eligibility.

#### 3.1.1. Inclusion Criteria

Patients were included in the study if they (1) were at least 21 years old, (2) underwent recent abdominal surgery for gastrointestinal perforation, malignancy, pancreatitis, intestinal obstruction, severe peritonitis, or liver transplant, and (3) had persistent postsurgical presentation of a systemic inflammatory response syndrome (SIRS) despite receiving broad spectrum antibiotics with intra-abdominal infection as the cause of sepsis, for example, peritonitis, colitis, and abscesses.

#### 3.1.2. Exclusion Criteria

Patients who (1) were less than 21 years of age, (2) had received any antifungal agents prior to SICU admission, and (3) had existing positive fungal cultures prior to ICU admission were excluded from the study.

#### 3.1.3. Treatment Allocation

**Figure 1:**
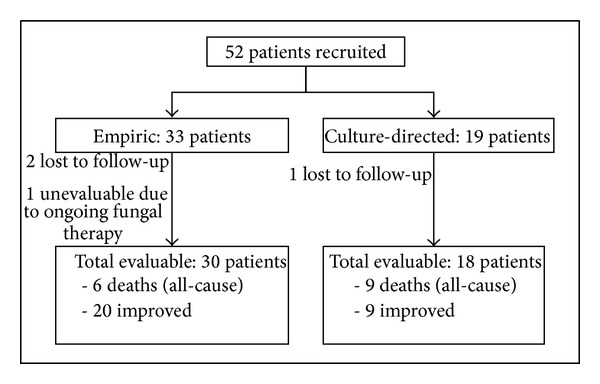
Flowchart depicting study enrolment and drop-out numbers.

As this was a noninterventional study, the choice, dose, and duration of antifungal and time to initiation of therapy were decided upon by patients' primary physicians. Depending on whether their antifungal treatment was empirical or culture-directed,patients who fulfilled the inclusion criteria were retrospectively allocated to two separate arms for analysis. Empiric antifungal therapy was defined as the receipt of any systemic antifungal drug, such as intravenous or oral azoles, intravenous echinocandins, or intravenous amphotericin B, prior to the first reporting of results for fungal cultures taken at the onset of sepsis. Culture-directed antifungal therapy was defined as the initiation of antifungal therapy after positive fungal culture results (Figure 1).

### 3.2. Data Collection and Evaluation

Patients' sociodemographics, comorbidities, underlying surgical condition, Acute Physiology and Chronic Health Evaluation II (APACHE II) and Sequential Organ Failure Assessment (SOFA) scores, routine laboratory values, microorganism culture results, and pertinent information from the case notes or patients' electronic medical records were systemically recorded on a standardized data collection form. All patients were followed up until death or discharge from hospital, whichever event occurred earlier. An infectious diseases physician blinded to patients' treatment group assessed patients' clinical outcomes to determine infectious-related and fungal-related mortality.

### 3.3. Microbiological Investigations and Definitions

Cultures of blood, urine, and intra-abdominal specimens (peritoneal fluid, pus, and tissue), respiratory tract (sputum and bronchoalveolar lavage), and skin and soft tissue (deep wound swabs) were performed whenever clinically indicated. Microorganisms were identified according to standard techniques in our hospital laboratory.

A positive fungal culture was considered significant if* Candida *was isolated from the blood, intra-abdominal fluid, intra-abdominal wound tissue, or urine specimens, with accompanying signs and symptoms of infection. Isolates of* Candida krusei *were assumed to be intrinsically resistant to fluconazole. A positive fungal urine culture was deemed significant if there are more than 10^5^ colonies of* Candida* per milliliter of urine with accompanying urine full examination microscopy elements (UFEME) analysis exhibiting elevated total white blood cells of more than 6 cells/mL, that is, pyuria and epithelial cells of less than 1 cell/mL.* Candida* isolated from the sputum was not considered significant as* Candida *pneumonia and lung abscess are very uncommon in nonneutropenic patients [21, 24].

### 3.4. Study Evaluable Endpoints

Mortality was evaluated as 30-day overall mortality, which was defined as death due to all causes within 30 days from the start of sepsis. Fungal-related mortality was defined as on-going sepsis with* Candida* isolated at any site (except sputum) upon death; or in the absence of positive fungal cultures, as on-going sepsis despite adequate antibiotics coverage for any coexisting bacterial infection and source control.

Length of SICU stay and hospitalization were measured from the time of SICU admission till death or discharge. Days to overall clinical improvement were calculated from the day of sepsis onset to the date when the subject showed an improvement in clinical parameters (normalization of or decreased trend in temperature, leukocyte counts, hemodynamic status, and any other signs indicative of sepsis), accompanied with either wound healing (removal of abdominal drains) and/or improved gut function (conversion from total parenteral nutrition to enteral feeds).

### 3.5. Statistical Analysis

Assuming a mortality rate of 40% in high-risk surgical patients [12] with delayed antifungal therapy and an absolute reduction of 20% mortality rate with early treatment, a sample size of 80 patients in each arm was required for a study power of 80% (alpha = 0.05, two-sided). Categorical comparisons were expressed as frequency distributions and evaluated using Fischer's exact test. Continuous variables were compared using nonparametric Wilcoxon-Mann-Whitney test. Kaplan-Meier survival curve was plotted to illustrate the difference in the time to overall clinical improvement for both arms and compared using the log-rank test. Multivariate logistic regression was performed to identify independent predictors of 30-day overall mortality in these high-risk surgical patient population. All statistical analyses were performed using STATA version 10.0.

## 4. Results

Out of 233 patients screened during the study period, 52 patients were started on antifungal therapy and included into the study. Of which, only 48 were eventually analyzed for study outcomes. Three patients were lost to follow-up as they defaulted further treatment and opted for hospital discharge whilst on antifungal therapy, total parenteral nutrition, and abdominal drains* in situ*. One patient was still on antifungal treatment at the point of analysis and all clinical outcomes could not be evaluated yet.

The sociodemographics, comorbidities, and severity of illness were fairly similar in both groups of patients, except that there were more males in the empiric arm. There were more patients with gastrointestinal perforations in the culture-directed group (Table 1).

### 4.1. Treatment Details

Two-thirds of the patients were given fluconazole (66.67%) and one-third was given echinocandins (31.25%). Amphotericin was used in a previous transplant recipient with blastoconidia in her blood cultures and the primary physician wanted to cover for Cryptococcus and Trichosporon in addition to* Candida*. Distribution of antifungal agents used and duration of therapy prescribed were fairly similar between both patient groups (Table 2), while the median days-to-initiation-of-therapy after onset of sepsis in the empiric treatment group was shorter than the culture-directed group by 4 days (*P* < 0.001).

### 4.2. Microbiological Results

The majority of the* Candida* isolated was unspeciated (42.9%), likely because they were from the sputum or urine, which made up more than one-third of the total number of samples cultured. For speciated* Candida*,* C. albicans *was the most common species (26.8%), followed by* C. tropicalis* (12.5%), and* C. glabrata* (10.7%). Overall, there were significantly more patients with positive fungal cultures in the culture-directed arm from sterile and nonsterile sites. On the contrary, there was no difference in the proportion of patients with concomitant bacterial infections between both arms (Table 3).

### 4.3. Primary and Secondary Outcomes

There were altogether 15 deaths in this study. Patients who received empiric therapy were 4 times less likely to experience 30-day all-cause mortality at 30 days than patients who had culture-directed treatment (OR: 0.25, 95% CI: 0.069 to 0.905; *P* = 0.03). Early empiric treatment also reduced fungal-related mortality but it did not significantly reduce length of hospital or ICU stay (Table 4).

**Figure 2:**
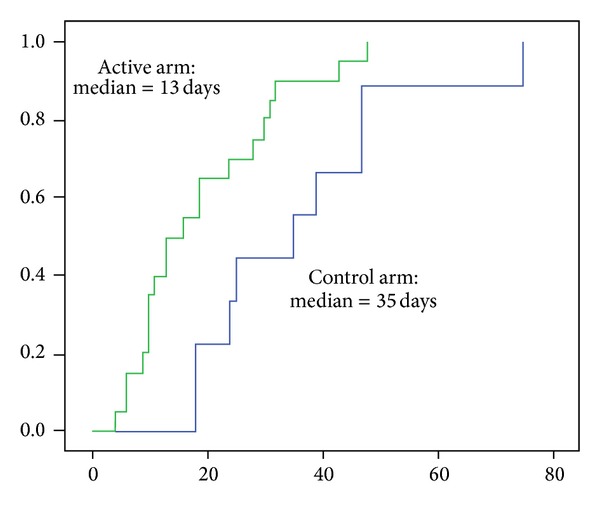
Kaplan-Meier survival estimate of time to clinical improvement.

Although there was no difference in the proportion of patients who demonstrated overall clinical improvement between the groups, a Kaplan-Meier survival plot (Figure 2) of the days to overall clinical improvement demonstrated that it was significantly shorter in patients who received early empiric therapy (*P* = 0.025). This observation was borne out in the analysis of the individual clinical parameters (Table 5).

After adjusting for possible confounders using multivariate logistic regression, patients who received early empiric antifungal therapy (adjusted OR 0.131, 95% CI 0.018 to 0.966; *P* = 0.046) and patients who underwent surgery for gastrointestinal malignancy (adjusted OR 0.038, 95% CI 0.002 to 0.892; *P* = 0.042) were found to have lower likelihood of 30-day overall mortality.

## 5. Discussion

There are three main routes of entry by which* Candida* gain access to the bloodstream: (a) via the gastrointestinal tract mucosal barrier; (b) via an intravascular catheter; and (c) from a localized focus of infection [25]. The early initiation of antifungal therapy in high-risk patients, especially those with prior colonization, can potentially limit the haematogenous spread of* Candida*. This in turn restricts widespread invasive candidiasis and decreases mortality risk. This concept is echoed in the existing body of evidence in the critically ill population which suggests that early antifungal therapy be used for patients with high risk (10% to 15%) for IC [26]. The IDSA's guidelines for the management of candidiasis also recommend that “empirical antifungal therapy should be considered in critically ill patients with risk factors for invasive candidiasis and no other known cause of fever” [21]. More recently, the European Society of Clinical Microbiology and Infectious Diseases (ESCMID) published their guidelines on the management of* Candida* diseases in nonneutropenic adult patients, in which the initiation of fluconazole or echinocandin for ICU patients who are persistently febrile but without microbiological evidence is marginally supported based on existing evidence [27].

In this study, the patient population selected was identified to be at particular risk for IC and most likely to benefit from early empiric antifungal therapy [25] or fungal prophylaxis as suggested in the ESCMID guidelines [25, 27]. We observed that starting early empiric treatment approach in this group of high-risk critically ill patients was significantly associated with reduced crude and fungal-related mortality rates and shorter time to overall clinical improvement. This was similar to that reported for 28 hospitals in Spain, where treatment initiated within 48 hours of the date when the first positive culture that was obtained was associated with higher probability of survival [28]. Morrell et al. also found that administration of antifungal agents 12 hours after sampling the first blood culture positive for* Candida* in patients with candidemia was an independent predictor of hospital mortality [13]. Their observations were confirmed by Garey et al. who showed that increased mortality was associated with delay in initiation of antifungal therapy [12]. The all-cause mortality rate reported for his patients who were treated on Day 1 (23.7%) and ≥3 days of culture (41.1%) was comparable to the patients in our study who were treated after a median of 1 day postsepsis (20%) and 5 days postsepsis (50%) in the empiric and culture-directed groups, respectively.

It was also noted that patients who started on early empiric antifungal treatment showed faster time to overall clinical improvement. However, there was no difference in the length of ICU stay or hospitalization. This was likely because patients had multiple factors affecting their ICU and hospital discharge such as need for mechanical ventilation or continuous renal replacement therapy.

Interestingly, from the regression analysis, patients who underwent abdominal surgery for malignancy were also more likely to survive beyond 30 days. This is biologically plausible as these surgeries were elective in nature and generally clean with little or no contamination of the intra-abdominal space. On the other hand, surgeries due to perforated gut and intestinal obstruction were often associated with gross contamination of the intra-abdominal space with the stomach contents. Patients who underwent recurrent operations for anastomotic leaks were also predisposed to intra-abdominal sepsis as a result of gut space contamination. The extent of gut contamination, as opposed to the reason for abdominal surgery, is probably the crucial factor affecting patients' survival in intra-abdominal sepsis as it indirectly reflects the bacterial and fungal burden in the site of infection.

Other than timing of antifungal therapy, the adequacy of antifungal agent used has been shown to have a significant impact on treatment efficacy. Adequacy of antifungal therapy is dependent on the choice, dose of the antifungal agent used, and types and susceptibility patterns of the* Candida* species involved. Zilberberg et al. evaluated the effect on inappropriate antifungal treatment in patients with candidal bloodstream infections and found that treatment delay of more than 24 hours or inadequate antifungal dose was independently associated with hospital mortality [29]. This observation was echoed in a large study by Parkins et al. [30], who reported that adequate empiric antifungal therapy was independently associated with a reduced risk of death (27 versus 46%, OR 0.46).

Despite little clinical data demonstrating clear superiority of echinocandins to other antifungal agents in invasive candidiasis, the IDSA guidelines for the management of candidiasis recommended that preference be given to an echinocandin when empirically treating a hemodynamically unstable or moderately-to-severely ill nonneutropenic patient for suspected candidiasis [21]. The rationale behind this is that echinocandins have excellent fungicidal activity against* Candida* species while azoles are fungistatic. A neutropenic murine invasive candidiasis model found that echinocandins reduce fungal burden in tissues more rapidly than fluconazole [31], thus suggesting better efficacy of echinocandins compared to azoles during early phase of candidemia.

As this study was noninterventional, the choice of antifungal agent prescribed was left to the primary physicians. Although not statistically significant, echinocandins were prescribed more often for empiric treatment while azoles were favored in culture-directed therapy. This reflected the primary physicians' general preference to cover more broadly for azole-non-susceptible species as well as for rapid reduction of fungal load early on in severe sepsis, in line with IDSA's recommendations. Fluconazole was most frequently used for culture-directed therapy as physicians generally felt more comfortable prescribing it based on susceptibility results. Furthermore, patients who were still alive when fungal culture results were back tended to be more stable clinically thus diminishing the need for rapid fungicidal activity.

Based on our study observations, using an echinocandin for empiric therapy may be a better option in patients with intra-abdominal sepsis with suspected IC as non-albicans* Candida* were sufficiently implicated. Furthermore, the heavy fungal burden at the site of infection necessitates the use of fungicidal agents.

### 5.1. Study Limitations

We were unable to recruit the target sample size within the study period due to the slow accrual rate in the 10-bedded SICU. Interestingly, we observed that the number of patients who received early empiric treatment increased over the study period. This pattern of use was probably due to increasing physician awareness and application of the empiric treatment approach recommended by the IDSA. As the number of patients who received culture-directed treatment declined, this study could not be feasibly continued.

Given the nonrandomized treatment allocation in this study, it was possible that treatment bias may have occurred when patients who received early empiric antifungal therapy were more aggressively treated than patients who were treated according to culture results. This may have confounded the reduced mortality observed in the empiric group, making it seem more significant than it really was.

The study outcomes may also be confounded by the difference in the number of positive fungal cultures between both groups. Unfortunately, further stratification by status of fungal cultures could not be reliably performed due to small sample size. The international classification system for fungal infections published by the European Organisation for Research and Treatment of Cancer/Invasive Fungal Infections Cooperative Group also could not be consistently applied to our study population [32]. Apart from proven fungal disease category, the patients in this study did not fulfil the host factors criteria to fit into “probable” or “possible” category.

Nevertheless, the diagnostic limitations of our study were minimized by ensuring that our patient cohort had the highest likelihood of invasive candidiasis according to the presence of risk factors such as use of total parenteral nutrition and broad-spectrum antibiotics, high APACHE scores, and gastrointestinal tract perforations [33, 34]. Purely colonized patients were excluded as all patients recruited had clinical manifestations of sepsis despite adequate broad-spectrum antibiotic use. Importantly, the clinical presentation for these patients was as described by IDSA and ESCMID guidelines.

Secondary outcomes such as clinical improvement also had subjective elements which may have caused data misclassification. This was minimized by blinding the ID physician who evaluated patients' clinical outcomes.

## 6. Conclusion

Despite the absence of strong evidence, the high mortality rates associated with delayed antifungal treatment supports early initiation of empiric antifungals. In our study, we observed that the patients with intra-abdominal sepsis, in whom empiric antifungal therapy was started earlier, had lower mortality and faster clinical recovery. Large-scale randomized controlled studies are warranted to determine the true effect of this approach on patients' survival. Future studies should also address the optimal duration of empiric antifungal therapy as well as the long-term effects of increased empiric antifungal use as current studies do not address potential emergence of less azole-susceptible strains. Until more data is available, the use of empiric antifungal treatment should be reserved for patients at the highest risk for IC where the benefit outweighs the risk of antifungal use.

## Figures and Tables

**Table 1:** Baseline demographics and medical conditions of patient cohort.

	Empiric (*n* = 30)	Culture-directed (*n* = 18)	*P* value		Empiric (*n* = 30)	Culture-directed (*n* = 18)	*P* value
Sociodemographics				Comorbidities			
Mean age (SD)	64 (11)	65 (14)	0.806	Median number of comorbidities (range)	3 (0–7)	3 (0–5)	0.528
Gender, *n* (%)			0.009	Respiratory	4 (13)	2 (11)	1.000
Male	20 (67)	5 (28)		Urology	1 (3)	0	1.000
Female	10 (33)	13 (72)		Cardiovascular	21 (70)	13 (72)	0.870
Race, *n* (%)			0.952	Gastrointestinal	17 (57)	4 (22)	0.020
Chinese	22 (73)	13 (72)		Endocrinology	18 (60)	11 (61)	0.939
Malay	2 (7)	1 (6)		Haematology	8 (27)	2 (11)	0.282
Indian	2 (7)	2 (11)		Liver impairment	9 (30)	4 (22)	0.740
Others	4 (13)	2 (11)		Renal impairment	11 (37)	7 (39)	0.878

Reason for surgery (*n*, %)							
Gastrointestinal Obstruction	5 (17)	3 (17)	1.000	Liver transplant	3 (10)	0	0.282
Gastrointestinal Malignancy	11 (37)	3 (17)	0.140	Peritonitis	1 (3)	2 (11.1)	0.547
Gastrointestinal Perforation	12 (40)	13 (72)	0.031	Pancreatitis	1 (3)	0	1.000

Severity of Illness							
Mean APACHE II Score (SD)	22 (6)	23 (8)	0.806	Mean SOFA score (SD)	9 (5)	9 (5)	0.890

**Table 2:** Details on antifungal therapy.

	Empiric (*n* = 30)	Culture-directed (*n* = 18)	*P* value
Choice of antifungal, *n* (%)			
Fluconazole	18 (60)	14 (78)	0.206
Caspofungin	9 (30)	3 (17)	0.493
Anidulafungin	3 (10)	0	0.282
Amphotericin	0	1 (6)	0.375
Mean total days of antifungal therapy (SD)	14 (9)	15 (7)	0.639
Median days to initiation of antifungal after sepsis (range)	1 (0–9)	5 (3–18)	<0.001

**Table 3:** Microbiological results.

	Empiric (*n* = 30)	Culture-directed (*n* = 18)	*P* value
Number of subjects with positive fungal cultures, *n* (%)	16 (53)	18 (100)	0.003
Number of subjects with significant fungal cultures, *n* (%)	12 (40)	14 (78)	0.011
Presence of concomitant bacterial infection, *n* (%)	28 (93)	15 (83)	0.349

Types of *Candida *species, *n* (%)			
*C. spp. *	10 (33)	14 (78)	0.003
*C. albicans *	7 (23)	8 (44)	0.127
*C. tropicalis *	3 (10)	4 (22)	0.400
*C. glabrata *	4 (13)	2 (11)	1.000
*C. krusei *	1 (3)	1 (6)	1.000
*C. parapsilosis *	1 (3)	0	1.000
*C. dubliniensis *	1 (3)	0	1.000

Location of isolated *Candida*, *n* (%)			
Sputum	5 (17)	3 (17)	1.000
Central venous catheter	0	1 (6)	0.375
Urine	8 (27)	7 (39)	0.376
Blood	3 (10)	4 (22)	0.400
Abdominal wound	3 (10)	5 (28)	0.132
Intraoperative wound	0	5 (28)	0.005
Peritoneal fluid	6 (20)	10 (56)	0.011
Drain	2 (7)	2 (11)	0.624

**Table 4:** Primary and secondary study outcomes.

	Empiric *n* = 30 (%)	Culture-directed *n* = 18 (%)	*P* value	Odds ratio (95% C.I.)
30-day overall mortality, *n* (%)	6 (20)	9 (50)	0.030	0.25 (0.069, 0.905)
30-day fungal-related mortality, *n* (%)	2 (7)	6 (33)	0.040	0.143 (0.025, 0.812)
Subjects with overall clinical improvement, *n* (%)	20 (67)	9 (50)	0.253	2.00 (0.605, 6.612)
Median length of hospital stay (range)	41.00 (5–161)	47.50 (7–125)	0.906	—
Median length of SICU stay (range)	9 (0–53)	10 (0–51)	0.925	—

**Table 5:** Time to improvement from sepsis (in days).

	Median time to improvement (range)			
	Overall clinical	Gut recovery	Drains removal	Normalization of leukocytes
Empiric	13 (4–48)	9 (0–48)	12.50 (3–38)	10 (2–48)
Culture-directed	35 (18–75)	18 (10–49)	31.50 (4–68)	26.50 (11–62)
*P* value	0.007	0.017	0.016	0.005
